# Two-stage association study to identify the genetic susceptibility of a novel common variant of rs2075290 in *ZPR1* to type 2 diabetes

**DOI:** 10.1038/srep29586

**Published:** 2016-07-14

**Authors:** Fanglin Guan, Yu Niu, Tianxiao Zhang, Songfang Liu, Lei Ma, Ting Qi, Jia Feng, Hong Zuo, Guohong Li, Xufeng Liu, Shujin Wang

**Affiliations:** 1 Health Science Center, Xi’an Jiaotong University, 76 Yanta West Road, Xi’an, China; 2 Department of Endocrinology and Metabolism, Ninth Hospital of Xi’an, 151 Eastern Section of Southern 2nd Ring Road, Xi’an, China; 3 Division of Biology & Biomedical Sciences, Washington University in Saint Louis, MO, USA

## Abstract

The SNP of rs964184 in *ZPR1* has recently been associated with type 2 diabetes mellitus (T2DM) in Japanese individuals. To comprehensively investigate the association of common variants in *ZPR1* with T2DM in Han Chinese individuals, we designed a two-stage case-control study of 3,505 T2DM patients and 6,911 unrelated healthy Han Chinese individuals. A total of 24 single nucleotide polymorphisms (SNPs) were genotyped, and single-SNP association, imputation and gender-specific association analyses were performed. To increase the coverage of genetic markers, we implemented imputation techniques to extend the number of tested makers to 280. A novel SNP, rs2075290, and the previously reported SNP, rs964184, were significantly associated with T2DM in the two independent datasets, and individuals harboring the CC genotype of rs2075290 and GG genotype of rs964184 exhibited higher levels of fasting plasma glucose (FPG) and blood hemoglobin A1c (HbA1c) than individuals of other genotypes. Additionally, haplotype analyses indicated that two haplotype blocks containing rs2075290 or rs964184 were also significantly associated with T2DM. In summary, these results suggest that *ZPR1* plays an important role in the etiology of T2DM, and this gene might be involved in abnormal glucose metabolism.

Type 2 diabetes mellitus (T2DM) is a major global health problem. Although the prevalence of the disease varies with age, sex, and population, the global age-standardized adult diabetes prevalence is >9.2%, and an estimated >347 million adults suffer from diabetes^1^. Accumulating evidence indicates that T2DM is a severe public health issue in China: approximately 113.9 million Chinese adults have diabetes, and 493.4 million Chinese adults have prediabetes^2^,^3^. Genetic susceptibility plays an important role in the development and/or progression of T2DM. Early epidemiological studies have identified a combination of multiple genetic and environmental factors involved in the pathogenesis of this disease^4^,^5^. Recently extensive genome-wide association studies (GWAS) for T2DM have been conducted worldwide, particularly in populations of European ancestry. Over 80 susceptibility loci have been identified, including *TCF7L2, SLC30A8, CDKN2A, CDKN2B, CDKAL1, KCNQ1, HHEX, LAMA1* and other genes^6^,^7^,^8^,^9^,^10^,^11^,^12^,^13^. A recent GWAS identified two susceptibility loci (*PTPRD* and *SRR*) for T2DM in Han Chinese individuals^14^. Another multistage GWAS examined the susceptibility to T2DM in Han Chinese women and identified a potential novel T2DM susceptibility locus at 13q31.1^15^. However, as a complex metabolic disorder subject to both genetic and environmental factors, the precise mechanisms underlying the development and progression of T2DM remain unknown^16^,^17^.

Recently, rs964184 in *ZPR1* was significantly associated with T2DM in a Japanese population, with the minor G allele representing a risk factor (with ORs at approximately 1.2) for this condition^18^. *ZPR1* (also known as *ZNF259*) contains 14 exons spanning a region of approximately 10 kb located on chromosome 11q23.3. This gene encodes a zinc-finger protein, ZPR1, which binds to the cytoplasmic tyrosine kinase domain of the epidermal growth factor receptor (EGFR) in quiescent cells and is essential for the normal functioning of the nucleoli during cell proliferation^19^,^20^. Additionally, the promoter site of *ZPR1* binds to peroxisome proliferator-activated receptor gamma (PPARG) proteins 1 and 2, which play a key role in insulin sensitivity and obesity^21^,^22^. PPARG 1 and 2 also bind to hepatocyte nuclear factor 4 alpha (HNF4α, nuclear receptor 2A1), which activates the genes involved in glucose and cholesterol metabolism in gastrointestinal and other digestive organs^23^. These results suggest that *ZPR1* might play a regulating function in the etiology of T2DM.

Because the underlying biological mechanisms of T2DM remain largely unknown, the contribution of *ZPR1* to T2DM has not been elucidated, despite the significant association between this gene and T2DM in Japanese individuals. Thus, to improve the current understanding of the role of *ZPR1* in the T2DM predisposition, it is important and necessary to investigate the consequences of genetic diversity in other ethnic populations. Currently, the role of *ZPR1* in T2DM risk in the Han Chinese population has not been previously assessed. Therefore, we conducted a two-stage case-control study to further determine the associations between *ZPR1* and the T2DM risk in Han Chinese individuals. A total of 3,505 newly diagnosed T2DM patients and 6,911 orthoglycemic healthy controls (1,854 cases and 3,595 controls in discovery stage, 1,651 cases and 3,316 controls in replication stage) were included. The aim of the present study was to evaluate the relationship of common variants in *ZPR1* with the increased risk of T2DM and explore the correlation of T2DM susceptibility loci with the levels of fasting plasma glucose (FPG) and blood hemoglobin A1c (HbA1c) in the Han Chinese population.

## Materials and Methods

### Subjects

The samples in the discovery stage were obtained from the city of Xi’an (The First Affiliated Hospital of Xi’an Jiaotong University; The Second Affiliated Hospital of Xi’an Jiaotong University; The Ninth Hospital of Xi’an) as representatives of the general population in Xi’an, and the samples in the replication stage were obtained from the city of Zhengzhou (The First Affiliated Hospital of Zhengzhou University) as representatives of the general population in Zhengzhou. From 2010 to 2014, all patients were recruited from the inpatient and outpatient clinical services (including health check-up centers) of these hospitals, and all unrelated healthy controls were recruited from the health check-up centers of these hospitals based on the selection criteria of frequency-matched age (±5 years) and gender of the patients. For the two-stage association study, we genotyped selected SNPs in 1,854 cases and 3,595 controls in the discovery stage. In the replication stage, we genotyped associated SNPs in additional samples from 1,651 cases and 3,316 controls. The details are shown in Table S1. In both stages, we determined through inquiry that all subjects were unrelated Han Chinese individuals with no migration history within the previous three generations. Trained interviewers administered a questionnaire to collect information on the demographic characteristics, family history, and disease history from every subject. T2DM was defined according to the criteria of the World Health Organization^24^ and subsequently confirmed by two experienced doctors. All patients were newly diagnosed with T2DM and had not previously taken any anti-diabetic medication. The patients showed FPG levels of ≥6.93 mmol/l and HbA1c contents of ≥6.5%. The healthy control individuals had FPG levels of <6.0 mmol/l, blood HbA1c contents of <6.1% and no history of DM or of receiving anti-diabetic medication. None of the healthy control individuals had first-degree relatives diagnosed with T2DM. The exclusion criteria for the study subjects were 1) organ failure; 2) severe cardiovascular/cerebrovascular diseases; 3) acute diabetic complications; 4) malignant tumors, severe injury/infections, pancreatic diseases or other metabolic/endocrinological diseases; 5) other types of diabetes; or 6) self-reported using anti-diabetic medication or medication that might cause raise in blood glucose levels. Additionally, individuals that met the following criteria were excluded from further analyses: (1) duplicate samples and (2) recorded evidence for relatedness. This study was performed in accordance with the ethical guidelines of the Declaration of Helsinki (version 2002) and was approved through the Ethics Committee of Xi’an Jiaotong University. All participants provided written informed consent.

### Serological testing and clinical characteristics of subjects

The demographic and clinical characteristics of gender, age, body mass index (BMI), diastolic blood pressure (DBP), and systolic blood pressure (SBP) were obtained through measurement and questionnaire (Table S1). Approximately 5 ml of venous blood was collected from each participant early in the morning after an overnight fast. The plasma glucose level was measured using the hexokinase-G6PD method (Denka Seiken, Niigata, Japan), which had an inter-assay coefficient of variation (CV) ≤3.0%. The HbA1C was assessed in the total blood. Specifically, the IFCC-standardized HbA1C was determined through High Performance Liquid Chromatography (HPLC) on a Tosoh G8 Analyzer using gradient ionic exchange. The inter-assay coefficients of variation were 0.7% and 1.2% for HbA1C values of 38.8 mmol/mol (5.7%) and 87.98 mmol/mol (10.2%), respectively. The levels of triglycerides (TG), total cholesterol (TC), low-density lipoprotein-cholesterol (LDL-C), and high-density lipoprotein-cholesterol (HDL-C) were measured using a Beckman Coulter AU5800 Auto-analyzer (Beckman Coulter, Cassina de’ Pecchi, Italy), according to the manufacturer’s instructions. These parameters are presented in Table S1, indicating that there were no statistical differences for the average age and gender distribution between cases and controls in the samples of either stage.

### SNP selection and genotyping

**Figure 1:**
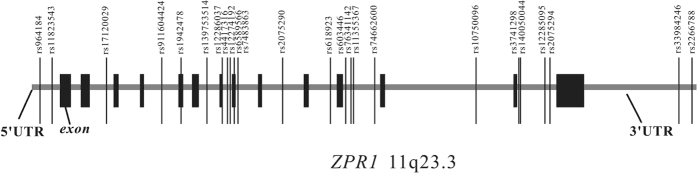
Distribution of the 24 SNPs across the *ZPR1* gene selected for the association analyses.

We searched for all SNPs in the *ZPR1* region with minor allele frequencies (MAF) ≥0.01 using the dbSNP database (Build 143) of NCBI (Build 38). Using this strategy, 24 SNPs were identified (rs964184, rs11823543, rs17120029, rs11604424, rs1942478, rs139753514, rs12286037, rs4417316, rs12274192, rs6589566, rs7483863, rs2075290, rs618923, rs603446, rs76341142, rs11355367, rs74662600, rs10750096, rs3741298, rs140050044, rs12285095, rs2075294, rs33984246, and rs2266788). These 24 SNPs, which completely covered the region of *ZPR1* (Fig. 1), were included in further analyses.

DNA was extracted from whole blood according to the standard protocol for the DNA Isolation Kit for Mammalian Blood (Tiangen Biotech Co., Ltd, Beijing, China). DNA was stored at −20 °C for SNP analyses. Genotyping was performed for all SNPs using the MassARRAY platform (Sequenom, San Diego, California, US). Briefly, the SNPs were genotyped using high-throughput, matrix-assisted laser desorption ionization–time-of-flight (MALDI–TOF) mass spectrometry. Next, the resulting spectra were processed using the MassARRAY Typer Analyzer software (Sequenom, San Diego, California, US), and genotype data were generated from the samples. For quality control, both the case and control status were blind during all genotyping processes. Meanwhile, 5% of random samples were repeated, and the results were 100% concordant.

### Statistical analyses

We implemented a logistic model using the genetic analysis software Plink v1.9 to investigate the association between genetic polymorphisms and disorder statuses in both the discovery and replication stages. The Bonferroni’s correction was applied to address the multiple comparison problem. Haploview v4.2 software was utilized to test the data for Hardy-Weinberg equilibrium (HWE) and calculate the minor allele frequency (MAF) for each marker in both stages. In addition, pair-wise LD analyses were applied to detect the inter-marker relationship based on D′ and *r*^2^ values using Haploview v4.2. The haplotype frequencies were estimated using GENECOUNTING v2.2, and haplotypic association analyses were performed for the common haplotypes (frequency > 0.01) using a likelihood ratio test followed by permutation testing. Imputations based on the discovery stage data were implemented using IMPUTE2 software with HapMap phase III CHB+JPT data and 1000 Genomes CHB data as two different reference panels. Association tests based on the imputed dosage data were conducted using SNPTEST v2 software. We utilized the parameter of “average certainty”, calculated in IMPUTE2, as the main indicator of imputation quality. The threshold of this indicator was selected by exploring the patterns of Q-Q plots based on the *P*-values of association analyses of multiple marker sets obtained using different certainty thresholds. The effects of T2DM-associated SNPs genotypes on the serum levels of FPG and HbA1c were evaluated after adjustments for potential confounding factors such as age, gender and BMI using analysis of variance, and conditional analyses to investigate independent effects of each of T2DM-associated SNPs were performed for genotype-phenotype correlation analyses. All tests were two-tailed, and a *P*-value of 0.05 was selected as a threshold for significance in all analyses. We used PGA v2.0 to perform the power calculations. The sample size used in the present study could detect SNP and haplotype associations with 85% and 81% power, respectively, at a false positive rate of 5% in the discovery stage.

### Ethics statements

All procedures were conducted in accordance with the ethical standards of the responsible committee on human experimentation (Committee on Human Rights Related to Research Involving Human Subjects, Xi’an Jiaotong University) and the Helsinki Declaration of 1975, revised in 2008.

## Results

### Allelic and genotypic association analyses

**Figure 2:**
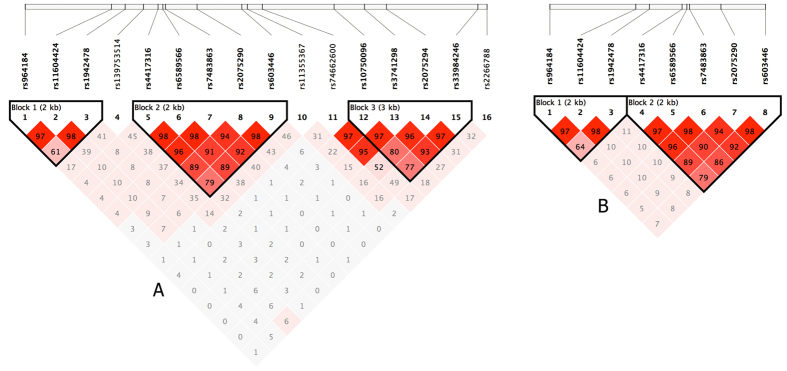
D structure based on two-stage data. The LD blocks are indicated as shaded matrices, and LD blocks 1 and 2 in the discovery stage (**A**) were confirmed in the replication stage (**B**).

Twenty-four SNPs in *ZPR1* were genotyped in the discovery dataset. The results of the single SNP-based association analyses of these SNPs in the discovery stage, including the Hardy–Weinberg equilibrium (HWE) test, are summarized in Tables 1 and S2. Polymorphisms were not observed for 8 SNPs (rs11823543, rs17120029, rs12286037, rs12274192, rs618923, rs76341142, rs140050044, and rs12285095). For the other 16 SNPs, we observed significant association signals for 2 SNPs (rs964184 and rs2075290; adjusted *P* = 0.001263 and 0.002735, respectively) (Table 1). Genotypic association analyses also confirmed the results with a similar pattern. The other 14 SNPs did not significantly differ in either genotype or allele distribution (Table S2). Based on the small effect sizes conferred through common alleles, which requires the use of large samples, the overall state of a given SNP is best summarized through association analyses of independent samples. Therefore, we performed single SNP association analyses for the two SNPs (rs964184 and rs2075290) with 6 other SNPs (rs11604424, rs1942478, rs4417316, rs6589566, rs7483863, and rs603446) within two different LD blocks (Fig. 2A) in the replication dataset. The significant association of the two SNPs (rs964184 and rs2075290) with T2DM was replicated (adjusted *P* = 0.002378 and 0.003876, respectively) (Table 1). The allelic or genotype distributions of the other 6 SNPs did not significantly differ (Table S2). In the combined analyses, the two SNPs remained strongly associated with T2DM (adjusted *P* = 6.007 × 10^−6^ and 1.654 × 10^−5^, respectively), and the odds ratios (OR) and 95% confidence interval (CI) indicated similar positive effects of the two associated SNPs on T2DM (Table 1). Age and gender information were included as two covariates in the fitting logistic, and conditional analyses suggested independent effects of each of the two SNPs in the genetic association analyses (Table 1). Additionally, rs964184 significantly deviated from HWE in the combined cases, consistent with the suggested association of the SNP with the disease^25^.

### Imputation and haplotypic association analyses

**Figure 3:**
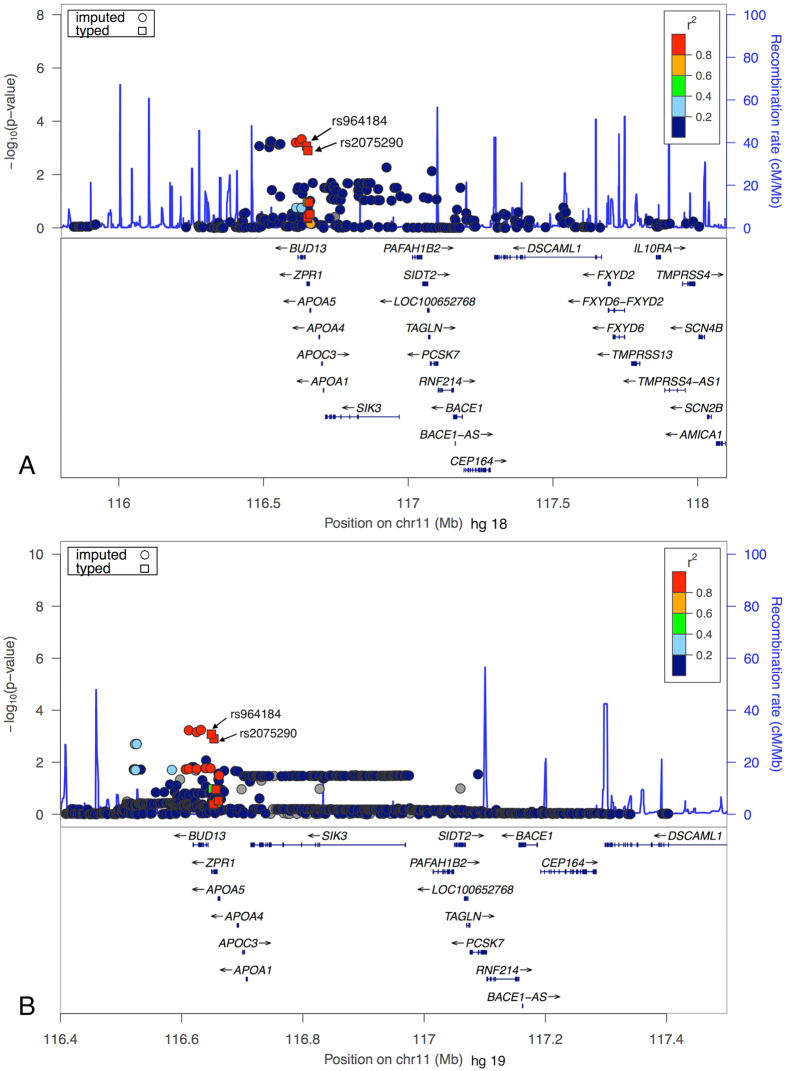
Regional association plots based on the imputed region covering the ZPR1 gene. Imputed SNPs are indicated as circles, and genotyped SNPs are indicated as squares. (**A**) Imputed using HapMap phase III CHB + JPT data. (**B**) Imputed using 1000 Genomes CHB data.

The imputations were implemented for a 5-Mb genomic region including *ZPR1* in chromosome 11. In both imputations with two different reference panels, we selected 0.8 as the average certainty threshold to exclude potential imputed SNPs with low accuracy. This threshold was selected after exploring the patterns of Q-Q plots based on the *P*-values of the association analyses of multiple marker sets obtained using different certainty thresholds in both imputations (Figure S1). Because we only focused on common SNPs, MAF ≥0.01 was applied as another filter criterion. After applying these filters, we successfully imputed 280 SNPs using the HapMap reference panel and 987 SNPs using the 1000 Genomes reference panel and examined these genes for association. We summarized 42 significant SNPs imputed with the HapMap reference panel, 179 significant SNPs imputed with the 1000 Genomes reference panel (P < 0.05), and 2 associated genotyped SNPs in Table S3. The results of the association analyses based on both imputations with two different reference panels are shown in Fig. 3.

Three LD blocks were identified using the data from the discovery stage, of which two blocks included rs964184 and rs2075290 (Fig. 2A). The two LD blocks were also constructed in the replication stage (Fig. 2B). Two regional LD structure plots are shown in Figure S2, indicating that several common SNPs are strongly associated with rs964184 and rs2075290. Subsequent haplotypic association analyses were used to test the two LD blocks mentioned above. As presented in Table 2, significant *P* values (global *P* = 0.038 and *P* < 0.001, respectively) were obtained in them. Some haplotypes in the two LD blocks were positively associated with T2DM. For example, the haplotypes GCG and GCT in block 1 and CAGCT in block 2 were significantly associated with T2DM as risk haplotypes. Because of the higher frequencies in healthy controls, the haplotype TAGTC in block 2 might protect against T2DM (Table 2). The ORs and 95% CI of these associated haplotypes also indicated a similar genetic effect on T2DM susceptibility (Table 2).

### Genotype-phenotype correlation of associated SNPs with FPG and HbA1c

To investigate the correlation of the 2 associated SNPs (rs964184 and rs2075290) in *ZPR1* with the blood glucose levels in T2DM patients and healthy controls, we performed multivariate analyses of FPG and HbA1c in each genotype group for both SNPs after adjustments for gender, age, and BMI. In both case and control groups, we observed significant differences in FPG and HbA1c for rs964184 and rs2075290 after the adjustments (*P* < 0.001). Specifically, the levels of FPG and HbA1c were highest in the GG genotype of rs964184 and CC genotype of rs2075290 (Table 3). Conditional analyses suggested that each of the two SNPs (rs964184 and rs2075290) exerted an independent effect in the genotype-phenotype association analyses, and the odds ratios (95% confidence interval) also indicated that the effects of the two associated SNPs on FPG and HbA1c were similar in cases and controls (Table 3).

## Discussion

In this two-stage study, two SNPs in *ZPR1*, rs964184 and rs2075290, were significantly associated with FPG and HbA1c. Specifically, in the two independent datasets, individuals harboring the GG genotype of rs964184 and the CC genotype of rs2075290 exhibited higher levels of FPG and HbA1c than individuals with other genotypes. The ORs and 95% CI also indicated the positive effects of the minor alleles of the two associated SNPs on T2DM. When comparing these results with those of Tokoro *et al*.^18^, we observed that rs964184 showed a significant association with T2DM, and the G allele was a risk allele in both studies. The OR of rs964184 was 1.23 in the present study compared with 1.242 according to Tokoro *et al*.^18^, and the risk allele frequency was much higher in the Japanese population (0.236 in our samples; 0.29 in the Tokoro *et al*.^18^ samples). However, rs2075290 showed no significant association with T2DM in the study of Tokoro *et al*.^18^. Indeed, analyses of only some SNPs are not sufficient to draw a conclusion. Our haplotype analyses also indicated that the rs964184 and rs2075290 were significantly associated with T2DM. Given the potential effect of the LD block on regulating gene transcription, we hypothesized that some SNPs might influence the expression of *ZPR1* in an unpredicted manner. Alternatively, these SNPs might coexist in LD with other undiscovered variants and participate with their regulation machinery to confer a risk for T2DM. This hypothesis is of interest for future studies. Despite the similarities in the general association patterns between the present study and that of Tokoro *et al*., these discrepancies might reflect differences in the ethnicity, genetic heterogeneity, sample size, type of polymorphism evaluated, gender and age of participants. However, these results are unlikely to be an artifact. First, significant signals were captured with both single SNP and haplotype-based analyses in two independent datasets. Moreover, conditional analyses indicated independent effects of each of the two SNPs (rs964184 and rs2075290) in the genetic association analyses and genotype-phenotype association analyses. Second, the problem of population stratification was avoided because all subjects were of Han descent and from the same geographical areas in each stage. Moreover, similar conclusions were reached in two different ethnic populations (Han Chinese and Japanese), reaffirming the association of *ZPR1* with T2DM.

Using imputation analyses^26^, we successfully imputed 280 SNPs with HapMap reference panel and 987 SNPs with the 1000 Genomes reference panel, and examined these variations for associations, increasing the marker set approximately 18 times (280 vs. 16) with HapMap reference data and 61 times (987 vs. 16) using 1000 Genomes reference data. When carefully assessing the regional association plots generated using the significant imputed SNPs as reference SNPs (Fig. 3 and Table S3), we identified a clustering pattern for *BUD13-ZPR1*. In a recent meta-analysis of 13 independent European ancestry studies, the SNPs of rs2075290 (*ZPR1*) and rs10790162 (*BUD13*) were significantly associated with metabolic syndrome^27^. Furthermore, inter-locus interactions among *BUD13-ZPR1* SNPs were also confirmed in a Southern Chinese population. These studies suggested that the top hits in imputation were not spurious. Indeed, the results of the present study could be biologically plausible, as accumulating evidence indicates that *ZPR1* plays a key role in the metabolism of glucose and lipids^21^,^22^,^23^. Previous studies have shown that rs964184 and rs2075290 in *ZPR1* were significantly associated with serum triglycerides and lipid levels^28^,^29^. Because an increase in the serum levels of triglycerides and lipids is an important risk factor for T2DM, multivariate analyses were performed in each genotype group for these associated SNPs after adjustments for gender, age, BMI, DBP, SBP, TG, TC, LDL-C, and HDL-C. The results suggested that *ZPR1* might be an independent risk factor for T2DM. However, because *APOA5* is adjacent to *ZPR1* (approximately 1.3 kb downstream of *APOA5*) and has been shown to significantly affect the development of T2DM in previous studies^30^,^31^, the effects of the two closely located genes are not easily separated. Notably, although bioinformatics-based approaches were used to assess the statistical associations between *ZPR1* polymorphisms and T2DM risk, whether the SNPs of rs964184 and rs2075290 are functional or acting as tags should be determined through additional functional studies, which might uncover fundamental characteristics of these variants.

Several limitations of the present study should be addressed. First, the hospital-based case-control study design often produced selection biases and information biases, which might be minimized by frequency matching for cases and controls and through adjustments for potential confounding factors in the multivariate analyses. Second, we did not exclude T2DM patients with hypertension or dyslipidemia, which would usually accompany and promote the development of T2DM. In addition, because of the retrospective nature of the study design, we did not have enough accurate information to conclusively rule out some potential risk confounders, such as smoking, alcohol consumption, lifestyle, and diet, which would play certain roles in accelerating the progress of T2DM and increasing the incidence rate of cardiovascular diseases. Given that the differences of these potential confounders between cases and controls would result from case or control selection, it is uncertain whether these findings could be extended to the general population. Thus, these results should be interpreted with caution, particularly when potential population stratification could not be completely excluded. Therefore, all of these findings should be considered preliminary, and additional follow-up studies are required to confirm these results in other ethnic populations. Third, although we detected associations between the SNPs within *ZPR1* and T2DM in the present study, we did not conduct an insulin sensitivity evaluation and regional fine mapping. T2DM is recognized as a complex and multifactorial disease, and single nucleotide alteration is insufficient for the prediction of the overall risk. Future studies include more genes and more SNPs, particularly more functional genes are required to ultimately unravel the effects of each genetic factor on the development of T2DM.

## Conclusion

To our knowledge, the present study represents the first large-scale genetic association study of *ZPR1* in Han Chinese individuals, and the findings regarding the SNP of rs2075290 in *ZPR1* are the first direct evidence linking the genetic susceptibility of common variants in *ZPR1* to T2DM with the levels of FPG and HbA1c. These results suggest that *ZPR1* might be involved in abnormal glucose metabolism and might serve as a therapeutic target and diagnostic molecular marker for assessing the susceptibility to T2DM. Given multiple variants that exert small effects and the molecular basis of the associations with respect to the complex network underlying the etiology and pathophysiology of T2DM, the present findings require replication in different ethnic populations to clarify the pathological mechanisms by which *ZPR1* plays a functional role in T2DM to utilize this gene as a target in clinical practice.

## Additional Information

**How to cite this article**: Guan, F. *et al*. Two-stage association study to identify the genetic susceptibility of a novel common variant of rs2075290 in *ZPR1* to type 2 diabetes. *Sci. Rep.* **6**, 29586; doi: 10.1038/srep29586 (2016).

## Supplementary Material

Supplementary Information

## Figures and Tables

**Table 1:** Results of single SNP association analyses.

**SNP (MAF)**	**Position**	**H-WE**	**Allelic** ***P*****-value**^1^	**OR**^2^ **95%CI**	**Genotypic** ***P*****-value**^1^	**OR**^3^ **95%CI**
The discovery stage						
rs964184 (G)	116,778,201		0.000827*	1.176	0.001743*	1.242
T2DM		0.130	0.001263**	1.069–1.292	0.003058**	1.093–1.411
CTRis		0.949	0.001604***		0.002832***	
rs2075290 (C)	116,782,580		0.001196*	1.162	0.002796*	1.182
T2DM		0.300	0.002135**	1.061–1.273	0.003046**	1.052–1.327
CTR		0.666	0.001715***		0.002341***	
The replication stage						
rs964184 (G)	116,778,201		0.001405*	1.177	0.001382*	1.271
T2DM		0.149	0.002378**	1.065–1.301	0.003509**	1.111–1.454
CTR		0.403	0.002128***		0.003247***	
rs2075290 (C)	116,782,580		0.001518*	1.168	0.002363*	1.232
T2DM		0.315	0.003876**	1.061–1.286	0.005913**	1.09–1.393
CTR		0.364	0.002389***		0.002305***	
Combined						
rs964184 (G)	116,778,201		4.146e–06*	1.175	1.62e–06*	1.252
T2DM		0.036	6.007e–06**	1.096***–***1.259	2.403e–06**	1.14***–***1.375
CTR		0.533	1.334e–05***		1.402e–05***	
rs2075290 (C)	116,782,580		5.908e–06*	1.159	3.482e–06*	1.215
T2DM		0.149	1.654e–05**	1.084***–***1.239	7.265e–06**	1.116***–***1.323
CTR		0.348	1.528e–05***		1.192e–05***	

MAF: minor allele; T2DM: type 2 diabetes mellitus; CTR: control; CI: confidence interval; OR: odds ratio.

^1^Significant *P* values are in italic bold, and *P*-value threshold corrected by Bonferroni’s correction are 0.0031 (0.05/16) in the discovery stage and 0.00625 (0.05/8) in the replication stage.

^2^OR refers to minor allele odds ratio in cases and controls.

^3^OR for genotypes in the additive model.

^*^Means *P* values before adjustments for covariants (gender, age and BMI).

^**^Means *P* values after adjustments for covariants (gender, age and BMI).

^***^Means *P* values of conditional analysis to investigate independent effects of each of the two SNPs.

**Table 2:** Common haplotype frequencies and association analyses T2DM: type 2 diabetes mellitus; CTR: control; OR: odds ratio; CI: confidence interval.

**Haplotype**	**Frequency in T2DM (%)**	**Frequency in CTR (%)**	***P*****-value**^1^	**OR** **95%CI**
Block 1 rs964184-rs11604424-rs1942478				
CTT	58.34	60.24	0.055	1.000
GCG	15.93	14.50	***0.030***	1.129 (1.006–1.266)
CCG	12.32	13.07	0.261	0.942 (0.822–1.081)
GCT	7.28	6.07	***0.021***	1.243 (1.043–1.481)
CCT	5.38	5.66	0.543	0.968 (0.795–1.180)
Block 2 rs4417316 -rs6589566-rs7483863-rs2075290 -rs603446				
CAGTC	64.31	64.52	0.825	1.000
TGACT	22.88	22.08	0.333	1.036 (0.939–1.144)
CAGTT	6.32	7.31	0.059	0.844 (0.774–1.001)
CAGCT	2.26	1.03	<***0.001***	2.339 (1.688–3.24)
TAGTC	1.63	2.33	***0.028***	0.669 (0.494–0.908)

Significant *P* values are indicated in bold italic font. Rare haplotypes are not shown, when the frequency is less than 1%.

Global *P* values are 0.038 and <0.001 in the Block 1 and Block 2, respectively, based on the comparison of the frequency distribution of all haplotypes for the combination of SNPs.

^1^Based on 10000 permutations.

**Table 3:** Correlation of associated SNPs with FPG and HbA1c.

**SNP**	**Genotype**	**FPG, mmol/L**	**HbA1c, %**	**FPG, mmol/L**	**HbA1c, %**
		T2DM group		Control group	
rs964184	CC	7.24 ± 0.02	6.92 ± 0.02	4.25 ± 0.01	4.76 ± 0.01
	CG	7.62 ± 0.03	7.05 ± 0.02	4.66 ± 0.01	4.94 ± 0.01
	GG	8.04 ± 0.07	7.51 ± 0.05	4.90 ± 0.03	5.27 ± 0.03
	*P*-value	**<*****0.001***	**<*****0.001***	**<*****0.001***	**<*****0.001***
	*P*-value*	**<*****0.001***	**<*****0.001***	**<*****0.001***	**<*****0.001***
rs2075290	TT	7.37 ± 0.03	6.92 ± 0.02	4.34 ± 0.01	4.81 ± 0.01
	TC	7.44 ± 0.02	7.04 ± 0.02	4.50 ± 0.01	4.87 ± 0.01
	CC	7.52 ± 0.06	7.15 ± 0.05	4.60 ± 0.03	4.96 ± 0.03
	*P*-value	**<*****0.001***	**<*****0.001***	**<*****0.001***	**<*****0.001***
	*P*-value*	**<*****0.001***	**<*****0.001***	**<*****0.001***	**<*****0.001***

FPG, fasting plasma glucose; HbA1c, blood hemoglobin A1c; OR: odds ratio; CI: confidence interval.

The data are presented as the means ± SE.

*P* values were adjusted for gender, age and BMI.

^*^Indicates the *P* values of conditional analysis to investigate independent effects of each of the two SNPs.

Significant *P* values are indicated in bold italic font.
