# Supplementary material for: Two-stage association study to identify the genetic susceptibility of a novel common variant of rs2075290 in ZPR1 to type 2 diabetes
Source: Sci Rep. 2016 Jul 14;6:29586. doi: 10.1038/srep29586 (PMC4944165; doi:10.1038/srep29586)
Supplement: Supplementary Information [file srep29586-s1.pdf]

Title: Two-stage association study to identify the genetic susceptibility of a novel common variant of rs2075290 in *ZPR1* to type 2 diabetes

Author names and affiliations: Fanglin Guan[a,*], Yu Niu[a,b,*], Tianxiao Zhang[c], Songfang Liu[b], Lei Ma[a,b], Ting Qi[b], Jia Feng[b], Hong Zuo[b], Guohong Li[b], Xufeng Liu[b] and Shujin Wang[b],

[a] Health Science Center, Xi'an Jiaotong University, 76 Yanta West Road, Xi'an, China;

[b] Department of Endocrinology and Metabolism，Ninth Hospital of Xi'an, 151 Eastern Section of Southern 2nd Ring Road, Xi'an, China;

[c] Division of Biology & Biomedical Sciences, Washington University in Saint Louis, MO, USA.

* These authors contributed equally to this work.

Corresponding author:

Fanglin Guan, M.D., Ph.D, Health Science Center, Xi'an Jiaotong University, Xi’an, China, 710061. Tel.: +86-29-82655474; Fax: +86-29-82655113, E-mail: fanglingguan@163.com

Shujin Wang, M.D., Department of Endocrinology and Metabolism，Ninth Hospital of Xi'an, 151 Eastern Section of Southern 2nd Ring Road, Xi'an, China, 710054. Tel.: +86-29-82322257; Fax: +86-29-82236643, E-mail: wangsj_paper@yeah.net

Table S1 The serological and clinic characteristics of T2DM in subjects

Characteristics	Cases	Controls	*P*-value (χ^2 or t)	Cases	Controls	*P*-value (χ^2 or t)
	Discovery stage			Replication stage		
Age (years)	49.76±11.31	50.04±11.59	0.396 (0.89)	51.72±10.48	51.86±11.33	0.673 (-0.42)
Range of age						
32-41 (discovery stage)	543	1054	NA			
31-40 (replication stage)				285	689	NA
42-51 (discovery stage)	533	1004	NA			
41-50 (replication stage)				373	770	NA
52-61 (discovery stage)	393	743	NA			
51-60 (replication stage)				512	922	NA
62-71 (discovery stage)	385	794	NA			
61-70 (replication stage)				482	935	NA
Gender (female/male) (%)	831 / 1023 (44.8%/55.2%)	1623 / 1972 (45.1%/54.9%)	0.820 (0.052)	749 / 902 (45.4%/54.6%)	1503 / 1813 (45.3%/54.7%)	0.978 (0.0007)
BMI (kg/m^2)	25.16±1.66	24.85±1.57	< 0.001 (-6.78)	25.17±1.62	24.79±1.60	< 0.001 (-7.89)
SBP (mm Hg)	138.69±9.46	122.88±7.37	< 0.001 (-62.77)	132.30±8.56	120.50±7.62	< 0.001 (-47.39)
DBP (mm Hg)	87.59±6.20	81.78±6.75	< 0.001 (-31.77)	79.09±10.59	78.71±8.71	0.197 (-1.29)
FPG (mmol/L)	7.43±1.10	4.42±0.62	< 0.001 (-109.2)	7.40±0.96	4.40±0.57	< 0.001 (-117.25)
HbA1c (%)	7.08±0.88	4.94±0.48	< 0.001 (-97.54)	6.92±0.76	4.74±0.47	< 0.001 (-107.27)
TC (mmol/L)	5.51±0.97	4.40±0.66	< 0.001 (-44.23)	5.44±1.05	4.38±0.64	< 0.001 (-37.94)
TG (mmol/L)	2.61±0.91	1.05±0.39	< 0.001 (-70.30)	2.48±0.80	1.06±0.37	< 0.001 (-68.71)
HDL-C (mmol/L)	0.98±0.18	1.56±0.29	< 0.001 (93.66)	0.89±0.17	1.59±0.26	< 0.001 (107.58)
LDL-C (mmol/L)	3.35±0.79	2.66±0.60	< 0.001 (-33.22)	3.33±0.81	2.58±0.62	< 0.001 (-33.25)

Data were shown as mean ±SD, except range of age and gender. NA, not applicable; T2DM, type 2 diabetes mellitus; BMI, body mass index; SBP, systolic blood pressure; DBP, diastolic blood pressure; FPG, fasting plasma glucose; TG, triglycerides; TC, total cholesterol; HDL-C, high density lipoprotein-cholesterol; LDL-C, low density lipoprotein-cholesterol; HbA1c, blood hemoglobin A1c.

Table S2 Allele and genotype frequency of single SNP association analysis in subjects

SNP	Position	H-WE	Allelic count (Freq. %)		*P*-value	Genotype count (Freq. %)			*P*-value
The discovery stage									
rs964184	116,778,201		C	G		CC	CG	GG	
T2DM		0.130	2833(76.4)	875(23.6)	***0.000827***	1094(59.01)	645(34.79)	115(6.2)	***0.001743***
CTR		0.949	5694(79.19)	1496(20.81)		2254(62.7)	1186(32.99)	155(4.31)	
rs11604424	116,780,399		T	C		TT	TC	CC	
T2DM		0.824	2191(59.09)	1517(40.91)	0.104563	645(34.79)	901(48.6)	308(16.61)	0.265257
CTR		0.923	4364(60.7)	2826(39.3)		1323(36.8)	1718(47.79)	554(15.41)	
rs1942478	116,780,747		T	G		TT	TG	GG	
T2DM		0.633	2647(71.39)	1061(28.61)	0.363737	949(51.19)	749(40.4)	156(8.41)	0.639372
CTR		0.842	5192(72.21)	1998(27.79)		1877(52.21)	1438(40)	280(7.79)	
rs139753514	116,781,214		A	- *		AA	A-	--	
T2DM		0.627	3058(82.47)	650(17.53)	0.134184	1264(68.18)	530(28.59)	60(3.24)	0.305047
CTR		0.967	6011(83.6)	1179(16.4)		2513(69.9)	985(27.4)	97(2.7)	
rs4417316	116,781,585		C	T		CC	CT	TT	
T2DM		0.843	2740(73.89)	968(26.11)	0.436174	1014(54.69)	712(38.4)	128(6.9)	0.733111
CTR		0.948	5263(73.2)	1927(26.8)		1927(53.6)	1409(39.19)	259(7.2)	
rs6589566	116,781,707		A	G		AA	AG	GG	
T2DM		0.865	2786(75.13)	922(24.87)	0.444071	1048(56.53)	690(37.22)	116(6.26)	0.745278
CTR		0.895	5450(75.8)	1740(24.2)		2067(57.5)	1316(36.61)	212(5.9)	
rs7483863	116,781,775		G	A		GG	GA	AA	
T2DM		0.885	2796(75.4)	912(24.6)	0.408455	1053(56.8)	690(37.22)	111(5.99)	0.708819
CTR		0.926	5473(76.12)	1717(23.88)		2082(57.91)	1309(36.41)	204(5.67)	
rs2075290	116,782,580		T	C		TT	TC	CC	
T2DM		0.300	2725(73.49)	983(26.51)	***0.001196***	1010(54.48)	705(38.03)	139(7.5)	***0.002796***
CTR		0.666	5487(76.31)	1703(23.69)		2089(58.11)	1309(36.41)	197(5.48)	
rs603446	116,783,719		C	T		CC	CT	TT	
T2DM		0.824	2499(67.39)	1209(32.61)	0.163427	840(45.31)	819(44.17)	195(10.52)	0.372657
CTR		0.997	4940(68.71)	2250(31.29)		1697(47.2)	1546(43)	352(9.79)	
rs11355367	116,783,861		A	- *		AA	A-	--	
T2DM		0.504	2361(63.67)	1347(36.33)	0.336856	745(40.18)	871(46.98)	238(12.84)	0.560698
CTR		0.918	4645(64.6)	2545(35.4)		1499(41.7)	1647(45.81)	449(12.49)	
rs74662600	116,784,243		G	A		GG	GA	AA	
T2DM		0.712	3170(85.49)	538(14.51)	0.256545	1357(73.19)	456(24.6)	41(2.21)	0.518370
CTR		0.844	6204(86.29)	986(13.71)		2678(74.49)	848(23.59)	69(1.92)	
rs10750096	116,786,072		A	C		AA	AC	CC	
T2DM		0.853	2911(78.51)	797(21.49)	0.112344	1144(61.7)	623(33.6)	87(4.69)	0.281638
CTR		0.968	5738(79.81)	1452(20.19)		2290(63.7)	1158(32.21)	147(4.09)	
rs3741298	116,786,845		T	C		TT	TC	CC	
T2DM		0.706	2065(55.69)	1643(44.31)	0.368432	571(30.8)	923(49.78)	360(19.42)	0.646116
CTR		0.926	4069(56.59)	3121(43.41)		1150(31.99)	1769(49.21)	676(18.8)	
rs2075294	116,787,406		G	T		GG	GT	TT	
T2DM		0.635	3270(88.19)	438(11.81)	0.433400	1444(77.89)	382(20.6)	28(1.51)	0.708849
CTR		0.863	6377(88.69)	813(11.31)		2829(78.69)	719(20)	47(1.31)	
rs33984246	116,789,734		A	G		AA	AG	GG	
T2DM		0.389	3260(87.92)	448(12.08)	0.472049	1437(77.51)	386(20.82)	31(1.67)	0.702685
CTR		0.683	6355(88.39)	835(11.61)		2811(78.19)	733(20.39)	51(1.42)	
rs2266788	116,789,970		A	G		AA	AG	GG	
T2DM		0.671	2732(73.68)	976(26.32)	0.302556	1010(54.48)	712(38.4)	132(7.12)	0.571167
CTR		0.869	5363(74.59)	1827(25.41)		2002(55.69)	1359(37.8)	234(6.51)	
The replication stage									
rs964184	116,778,201		C	G		CC	CG	GG	
T2DM		0.149	2529(76.59)	773(23.41)	***0.001405***	979(59.3)	571(34.59)	101(6.12)	***0.001382***
CTR		0.403	5265(79.39)	1367(20.61)		2082(62.79)	1101(33.2)	133(4.01)	
rs11604424	116,780,399		T	C		TT	TC	CC	
T2DM		0.755	1942(58.81)	1360(41.19)	0.217119	568(34.4)	806(48.82)	277(16.78)	0.455410
CTR		0.951	3986(60.1)	2646(39.9)		1197(36.1)	1592(48.01)	527(15.89)	
rs1942478	116,780,747		T	G		TT	TG	GG	
T2DM		0.544	2334(70.68)	968(29.32)	0.338147	830(50.27)	674(40.82)	147(8.9)	0.523528
CTR		0.843	4749(71.61)	1883(28.39)		1698(51.21)	1353(40.8)	265(7.99)	
rs4417316	116,781,585		C	T		CC	CT	TT	
T2DM		0.526	2446(74.08)	856(25.92)	0.690301	901(54.57)	644(39.01)	106(6.42)	0.744473
CTR		0.809	4888(73.7)	1744(26.3)		1804(54.4)	1280(38.6)	232(7)	
rs6589566	116,781,707		A	G		AA	AG	GG	
T2DM		0.418	2474(74.92)	828(25.08)	0.693886	933(56.51)	608(36.83)	110(6.66)	0.828947
CTR		0.746	4993(75.29)	1639(24.71)		1883(56.79)	1227(37)	206(6.21)	

rs7483863	116,781,775		G	A		GG	GA	AA	
T2DM		0.848	2504(75.83)	798(24.17)	0.693321	948(57.42)	608(36.83)	95(5.75)	0.923932
CTR		0.850	5053(76.19)	1579(23.81)		1923(57.99)	1207(36.4)	186(5.61)	
rs2075290	116,782,580		T	C		TT	TC	CC	
T2DM		0.315	2434(73.71)	868(26.29)	***0.001518***	905(54.82)	624(37.8)	122(7.39)	***0.002363***
CTR		0.364	5081(76.61)	1551(23.39)		1937(58.41)	1207(36.4)	172(5.19)	
rs603446	116,783,719		C	T		CC	CT	TT	
T2DM		0.831	2215(67.08)	1087(32.92)	0.212515	741(44.88)	733(44.4)	177(10.72)	0.448165
CTR		0.923	4531(68.32)	2101(31.68)		1549(46.71)	1433(43.21)	334(10.07)	

T2DM: type 2 diabetes mellitus; CTR: control.
* means allele deletion.
Significant *P* values are in italic bold, and *P*-value threshold corrected by Bonferroni correction are 0.0031 (0.05/16) in the discovery stage and 0.00625 (0.05/8) in the replication stage.

Table S3 The association test results of imputed and genotyped SNPs with significance

SNP	Position	Type	Allele A	Allele B	MAF	*P*-value
Imputation by HapMap CHB+JBT as reference panel						
rs3825041	116760991	imputed	C	T	0.1498	4.74E-04
rs481843	116655150	imputed	C	T	0.0877	5.60E-04
rs480823	116655013	imputed	C	T	0.0877	5.81E-04
rs480878	116652137	imputed	A	G	0.0880	5.85E-04
rs180326	116753987	imputed	G	T	0.1620	6.05E-04
rs180349	116741111	imputed	A	T	0.1609	6.31E-04
rs12799766	116687711	imputed	A	G	0.0898	7.13E-04
rs17092638	116685520	imputed	C	G	0.0915	7.40E-04
rs12805061	116682308	imputed	A	G	0.0917	7.53E-04
rs964184	116778201	typed	C	G	0.2176	8.47E-04
rs498736	116614617	imputed	C	G	0.1004	8.57E-04
rs499790	116649022	imputed	C	T	0.0842	9.39E-04
rs2075290	116782580	typed	C	T	0.2465	1.26E-03
rs723953	117054262	imputed	A	C	0.0999	5.40E-03
rs1263499	117210993	imputed	A	G	0.0587	7.43E-03
rs11216164	116863829	imputed	A	G	0.0166	2.03E-02
rs11600380	116799466	imputed	C	T	0.0111	2.07E-02
rs638392	116989740	imputed	A	T	0.0511	2.09E-02
rs569568	116986684	imputed	C	T	0.0511	2.09E-02
rs10502223	116999111	imputed	C	T	0.0510	2.10E-02
rs6589579	116918919	imputed	A	C	0.0509	2.11E-02
rs2513091	116974822	imputed	A	G	0.0510	2.11E-02
rs2513092	116973563	imputed	C	T	0.0510	2.12E-02
rs587703	116956849	imputed	A	G	0.0510	2.12E-02
rs522848	116921729	imputed	C	T	0.0509	2.15E-02
rs1263160	116873731	imputed	C	T	0.0545	2.81E-02
rs10892052	116963597	imputed	A	G	0.0924	2.87E-02
rs599320	116869943	imputed	C	G	0.0518	2.90E-02
rs11216129	116749540	imputed	A	C	0.0468	3.00E-02
rs623908	116769652	imputed	A	G	0.0716	3.11E-02
rs632153	116839523	imputed	G	T	0.0550	3.13E-02
rs28989469	117309465	imputed	C	T	0.0310	3.13E-02
rs11216267	117081676	imputed	C	T	0.0986	3.14E-02
rs10892072	117089597	imputed	A	G	0.0987	3.14E-02
rs614944	116846947	imputed	C	T	0.0527	3.16E-02
rs640411	116854587	imputed	C	G	0.0529	3.21E-02
rs509712	116857313	imputed	A	G	0.0529	3.24E-02
rs598503	116853488	imputed	C	T	0.0529	3.24E-02
rs10892063	117025439	imputed	A	C	0.0968	3.50E-02
rs11216126	116746524	imputed	A	C	0.0495	3.76E-02
rs10892053	116986287	imputed	C	T	0.0955	3.80E-02
rs10502221	116956154	imputed	C	T	0.0952	3.82E-02
rs496958	116658159	imputed	C	T	0.0658	3.82E-02
rs7120706	116959371	imputed	C	T	0.0961	4.04E-02
rs7120963	116959545	imputed	C	T	0.0961	4.04E-02
rs482371	116881447	imputed	C	T	0.0877	4.06E-02
Imputation by 1000 genomes CHB as reference panel						
rs3825041	116631707	imputed	T	C	0.1686	5.67E-04
rs180349	116611827	imputed	A	T	0.2051	5.92E-04
rs180326	116624703	imputed	G	T	0.2051	6.93E-04
rs964184	116648917	typed	G	C	0.2176	8.31E-04
rs2075290	116653296	typed	C	T	0.2465	1.25E-03
rs481843	116525867	imputed	C	T	0.0994	1.95E-03
rs480823	116525730	imputed	T	C	0.0995	1.99E-03
rs480878	116522854	imputed	G	A	0.1000	1.99E-03
rs623908	116640368	imputed	A	G	0.1386	8.46E-03

rs11216126	116617240	imputed	A	C	0.0992	1.53E-02
rs11216129	116620256	imputed	C	A	0.0971	1.55E-02
rs10790162	116639104	imputed	A	G	0.1685	1.67E-02
rs6589565	116640237	imputed	A	G	0.1684	1.68E-02
rs2160669	116647607	imputed	C	T	0.1839	1.69E-02
rs9326246	116611733	imputed	C	G	0.1745	1.80E-02
rs7930786	116624727	imputed	C	G	0.1744	1.81E-02
rs523980	116523306	imputed	A	T	0.0850	1.85E-02
rs141584077	116523304	imputed	A	T	0.0850	1.85E-02
rs533668	116521135	imputed	T	G	0.1002	1.87E-02
rs1974718	116606766	imputed	G	A	0.1768	1.88E-02
rs1558860	116607368	imputed	A	C	0.1768	1.88E-02
rs1558861	116607437	imputed	C	T	0.1768	1.88E-02
rs549125	116520926	imputed	C	G	0.1003	1.88E-02
rs118175510	116532548	imputed	T	C	0.0917	1.90E-02
rs573985	116521316	imputed	G	A	0.0961	1.94E-02
rs563796	116522111	imputed	A	G	0.1002	1.94E-02
rs563917	116522156	imputed	C	T	0.1001	1.94E-02
rs492182	116522164	imputed	G	A	0.1001	1.95E-02
rs562756	116521975	imputed	G	C	0.0961	1.96E-02
rs151007118	116583864	imputed	G	T	0.1104	1.96E-02
rs575906	116521558	imputed	A	G	0.0961	1.96E-02
rs528732	116523844	imputed	G	A	0.0999	1.98E-02
rs567599	116522565	imputed	G	T	0.1001	1.98E-02
rs567576	116522559	imputed	A	G	0.1001	1.98E-02
rs528647	116523821	imputed	C	T	0.0999	1.98E-02
rs3017778	116523931	imputed	C	T	0.0999	1.98E-02
rs3017779	116523988	imputed	C	T	0.0999	1.99E-02
rs202191031	116522976	imputed	AAGTGG	A	0.1000	1.99E-02
rs138535309	116522970	imputed	CTT	C	0.1000	1.99E-02
rs499563	116522961	imputed	C	T	0.1000	1.99E-02
rs3017237	116522625	imputed	G	C	0.1001	1.99E-02
rs480028	116522770	imputed	C	G	0.1000	1.99E-02
rs483747	116523167	imputed	T	C	0.1000	1.99E-02
rs34485603	116526032	imputed	CCT	C	0.0994	2.01E-02
rs565607	116522330	imputed	C	T	0.1001	2.01E-02
rs485638	116523373	imputed	G	C	0.1000	2.01E-02
rs66505542	116623213	imputed	TA	T	0.2641	2.07E-02
rs11600380	116670182	imputed	T	C	0.0407	2.13E-02
rs2727790	116688383	imputed	G	A	0.0506	2.18E-02
rs5094	116693213	imputed	G	A	0.0469	2.26E-02
rs34089864	116660768	imputed	G	A	0.0980	2.75E-02
rs78068721	117088882	imputed	C	T	0.0314	2.89E-02
rs377283819	116887039	imputed	GA	G	0.0456	3.14E-02
11:116880696	116880696	imputed	A	C	0.0316	3.21E-02
rs143226700	116971807	imputed	AG	A	0.0640	3.21E-02
rs2072560	116661826	imputed	T	C	0.1761	3.21E-02
rs56257632	116959601	imputed	T	C	0.0640	3.22E-02
rs75830246	116968440	imputed	T	C	0.0641	3.22E-02
rs73576687	116970285	imputed	G	A	0.0640	3.22E-02
rs7939209	116969416	imputed	T	C	0.0641	3.23E-02
rs7939303	116969439	imputed	T	C	0.0641	3.23E-02
rs73576682	116968225	imputed	A	G	0.0641	3.23E-02
rs73576686	116970006	imputed	T	A	0.0640	3.23E-02
rs59330954	116958158	imputed	C	CA	0.0640	3.24E-02
rs7112542	116960078	imputed	G	A	0.0640	3.24E-02
rs118155427	116975084	imputed	G	C	0.0642	3.26E-02
rs3016611	116847584	imputed	G	A	0.0636	3.26E-02
rs80137897	116954760	imputed	T	C	0.0639	3.27E-02
rs2513092	116844279	imputed	T	C	0.0636	3.27E-02

rs147116575	116783725	imputed	T	G	0.0318	3.27E-02
rs2513091	116845538	imputed	A	G	0.0636	3.27E-02
rs7121475	116951615	imputed	G	A	0.0639	3.27E-02
rs77564847	116955831	imputed	T	C	0.0639	3.28E-02
rs73576652	116950567	imputed	G	A	0.0639	3.28E-02
rs73576656	116953604	imputed	C	T	0.0639	3.29E-02
rs73576658	116953784	imputed	G	A	0.0639	3.29E-02
rs79536557	116963969	imputed	A	G	0.0641	3.29E-02
rs59752757	116950278	imputed	A	C	0.0639	3.30E-02
rs73576634	116944304	imputed	G	T	0.0639	3.30E-02
rs2441218	116854580	imputed	T	G	0.0636	3.30E-02
rs7124649	116948640	imputed	T	C	0.0639	3.30E-02
rs537431	116856179	imputed	T	C	0.0636	3.30E-02
rs7108830	116948761	imputed	G	A	0.0639	3.30E-02
rs56188860	116948173	imputed	T	A	0.0639	3.30E-02
rs59839888	116949853	imputed	T	C	0.0639	3.30E-02
rs73594187	116915881	imputed	T	C	0.0638	3.30E-02
rs140772203	116915330	imputed	A	AG	0.0638	3.31E-02
rs73576637	116945074	imputed	A	G	0.0639	3.31E-02
rs56125819	116961617	imputed	G	A	0.0641	3.31E-02
rs73594185	116915229	imputed	C	T	0.0638	3.31E-02
rs57787606	116942479	imputed	G	A	0.0639	3.31E-02
rs73594158	116896441	imputed	C	T	0.0637	3.31E-02
rs61244196	116961510	imputed	G	A	0.0641	3.31E-02
rs73594169	116899617	imputed	C	T	0.0637	3.31E-02
rs73594162	116896811	imputed	C	T	0.0637	3.31E-02
rs58848374	116938655	imputed	G	A	0.0638	3.31E-02
rs569568	116857400	imputed	T	C	0.0637	3.31E-02
rs17120275	116939071	imputed	T	C	0.0638	3.32E-02
rs75949389	116892626	imputed	A	G	0.0637	3.32E-02
rs111641976	116941382	imputed	G	A	0.0639	3.32E-02
rs7114173	116928336	imputed	T	C	0.0638	3.32E-02
rs73594168	116899414	imputed	C	T	0.0637	3.32E-02
rs7113829	116890259	imputed	C	T	0.0637	3.32E-02
rs622302	116831071	imputed	A	G	0.0634	3.32E-02
rs150734126	116880018	imputed	T	C	0.0637	3.32E-02
rs145339787	116921143	imputed	T	C	0.0638	3.32E-02
rs139130245	116920936	imputed	CG	C	0.0638	3.32E-02
rs10502225	116939724	imputed	A	T	0.0638	3.32E-02
rs73594164	116897613	imputed	T	G	0.0637	3.32E-02
rs145133311	116886516	imputed	C	T	0.0637	3.32E-02
rs73594141	116882563	imputed	C	T	0.0637	3.33E-02
rs73594175	116909994	imputed	A	G	0.0638	3.33E-02
rs1261706	116819991	imputed	T	A	0.0633	3.33E-02
rs73576603	116917885	imputed	G	A	0.0638	3.33E-02
rs61582023	116911784	imputed	A	G	0.0638	3.33E-02
rs638392	116860456	imputed	T	A	0.0637	3.33E-02
rs73594177	116910320	imputed	G	T	0.0638	3.33E-02
rs142693807	116904176	imputed	G	A	0.0637	3.33E-02
rs623423	116861519	imputed	G	C	0.0637	3.33E-02
rs560897	116862087	imputed	C	T	0.0637	3.33E-02
rs73594181	116910427	imputed	T	A	0.0638	3.33E-02
rs3133352	116818187	imputed	G	A	0.0633	3.33E-02
rs59647391	116900956	imputed	A	G	0.0637	3.33E-02
rs73594172	116902631	imputed	A	T	0.0637	3.34E-02
rs79286504	116817980	imputed	A	T	0.0633	3.34E-02
rs607756	116862718	imputed	T	C	0.0637	3.34E-02
rs1982679	116936488	imputed	G	A	0.0638	3.34E-02
rs34661549	116817084	imputed	T	TA	0.0633	3.34E-02
rs79340485	116910454	imputed	C	T	0.0638	3.34E-02

rs10502223	116869827	imputed	T	C	0.0637	3.34E-02
rs113712143	116817785	imputed	G	A	0.0633	3.34E-02
rs73576612	116929666	imputed	T	C	0.0638	3.34E-02
rs17120271	116935363	imputed	C	G	0.0638	3.34E-02
rs2513099	116811057	imputed	T	C	0.0633	3.34E-02
rs10616066	116826609	imputed	CAT	C	0.0634	3.34E-02
rs587703	116827565	imputed	A	G	0.0634	3.34E-02
rs649084	116815769	imputed	G	A	0.0633	3.34E-02
rs2921654	116797734	imputed	C	T	0.0633	3.34E-02
rs73594128	116866609	imputed	T	C	0.0637	3.34E-02
rs111426457	116931087	imputed	C	G	0.0638	3.35E-02
rs497653	116828484	imputed	T	C	0.0634	3.35E-02
rs7946101	116934869	imputed	G	A	0.0638	3.35E-02
rs547919	116800154	imputed	A	T	0.0633	3.35E-02
rs585750	116821719	imputed	A	G	0.0634	3.35E-02
rs522848	116792445	imputed	T	C	0.0633	3.35E-02
rs142743225	116795160	imputed	T	TTTTTG	0.0633	3.35E-02
rs17120265	116932615	imputed	C	G	0.0638	3.36E-02
rs6589579	116789635	imputed	C	A	0.0633	3.36E-02
rs1979023	116778180	imputed	C	T	0.0632	3.37E-02
rs557331	116734688	imputed	G	A	0.0629	3.37E-02
rs598503	116724204	imputed	T	C	0.0628	3.38E-02
rs541586	116729196	imputed	T	C	0.0629	3.38E-02
rs57301539	116751978	imputed	T	TTG	0.0631	3.38E-02
rs628418	116718366	imputed	C	G	0.0626	3.38E-02
rs595137	116771356	imputed	C	T	0.0632	3.39E-02
rs1263160	116744447	imputed	T	C	0.0631	3.39E-02
rs676234	116722171	imputed	A	G	0.0628	3.39E-02
rs472111	116716869	imputed	G	A	0.0626	3.40E-02
rs614944	116717663	imputed	T	C	0.0626	3.40E-02
rs1263162	116705021	imputed	T	A	0.0621	3.40E-02
rs1241550	116726651	imputed	T	C	0.0629	3.40E-02
rs10750099	116788699	imputed	A	C	0.0633	3.41E-02
rs675834	116737736	imputed	T	A	0.0629	3.41E-02
rs509712	116728029	imputed	A	G	0.0629	3.41E-02
rs599320	116740659	imputed	G	C	0.0629	3.41E-02
rs11420452	116716006	imputed	G	GA	0.0625	3.42E-02
rs576262	116764436	imputed	C	G	0.0632	3.42E-02
rs5132	116702778	imputed	C	T	0.0620	3.43E-02
rs640411	116725303	imputed	G	C	0.0628	3.43E-02
rs113749676	116712442	imputed	C	T	0.0623	3.44E-02
rs632153	116710239	imputed	G	T	0.0623	3.44E-02
rs5081	116706346	imputed	A	T	0.0621	3.45E-02
rs2921655	116836548	imputed	G	C	0.0595	3.53E-02
rs581122	116838615	imputed	C	T	0.0595	3.55E-02
rs667119	116835883	imputed	A	T	0.0595	3.56E-02
rs541496	116835362	imputed	C	T	0.0595	3.57E-02
rs375816794	116731466	imputed	T	TCATCG	0.0591	3.64E-02
rs493101	116833669	imputed	G	A	0.0697	4.23E-02
rs148759216	116660554	imputed	C	CCT	0.0993	4.45E-02
11:116597394	116597394	imputed	G	A	0.0102	4.64E-02
rs2098454	116634267	imputed	A	T	0.0102	4.90E-02

MAF: minor allele frequency

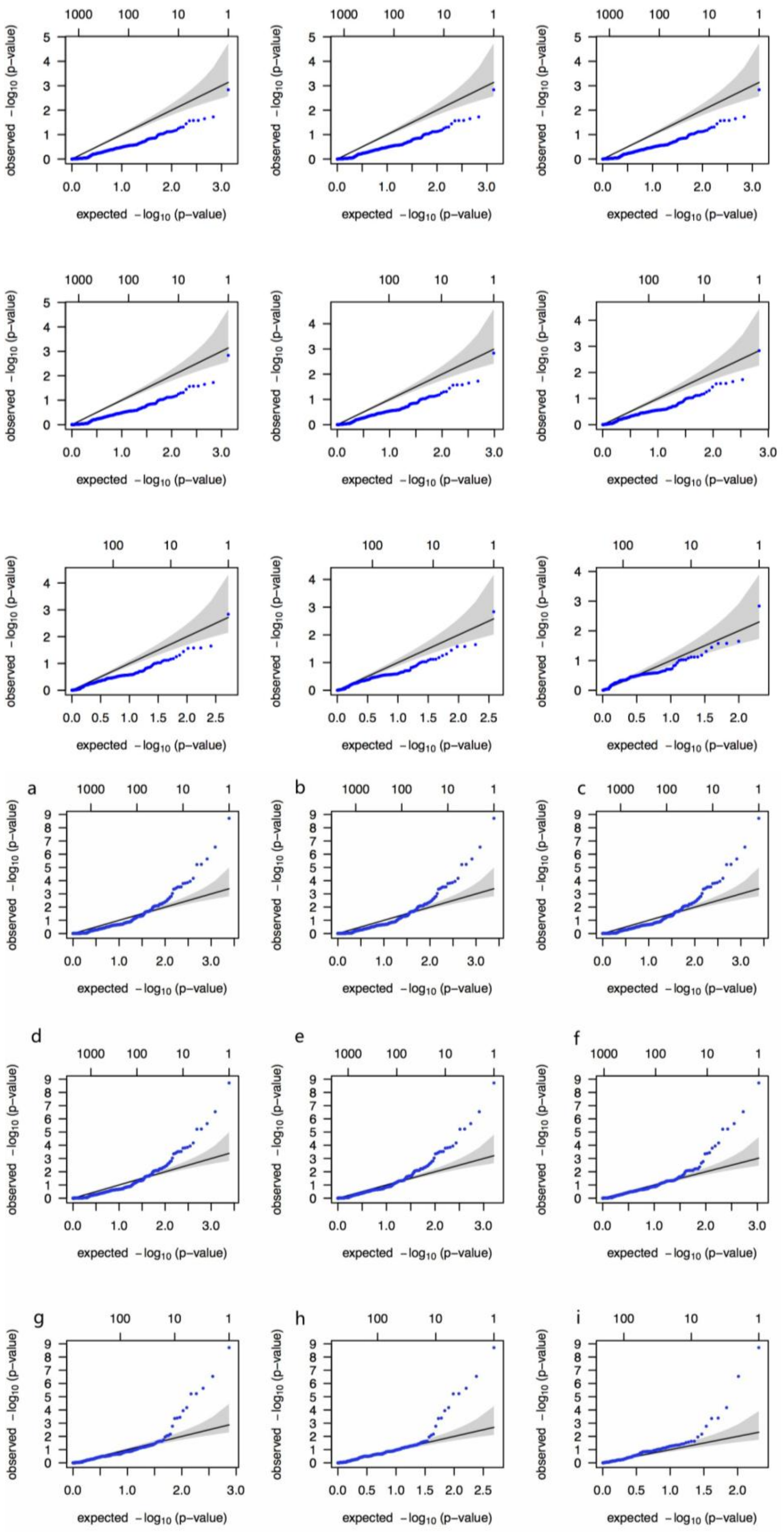

Figure S1. The Q-Q plots made by association test results based on marker set filter by different certainty thresholds with the reference panel of HapMap CHB+JPT (A) and 1000 genomes CHB (B) . The certainty thresholds used in a, b, c, d, e, f, g, h, i were 0.1, 0.2, 0.3, 0.4, 0.5, 0.6, 0.7, 0.8, 0.9 respectively. No significant deviance from expected line could be found when the certainty threshold was chosen as 0.8 in both imputations with different reference panels.

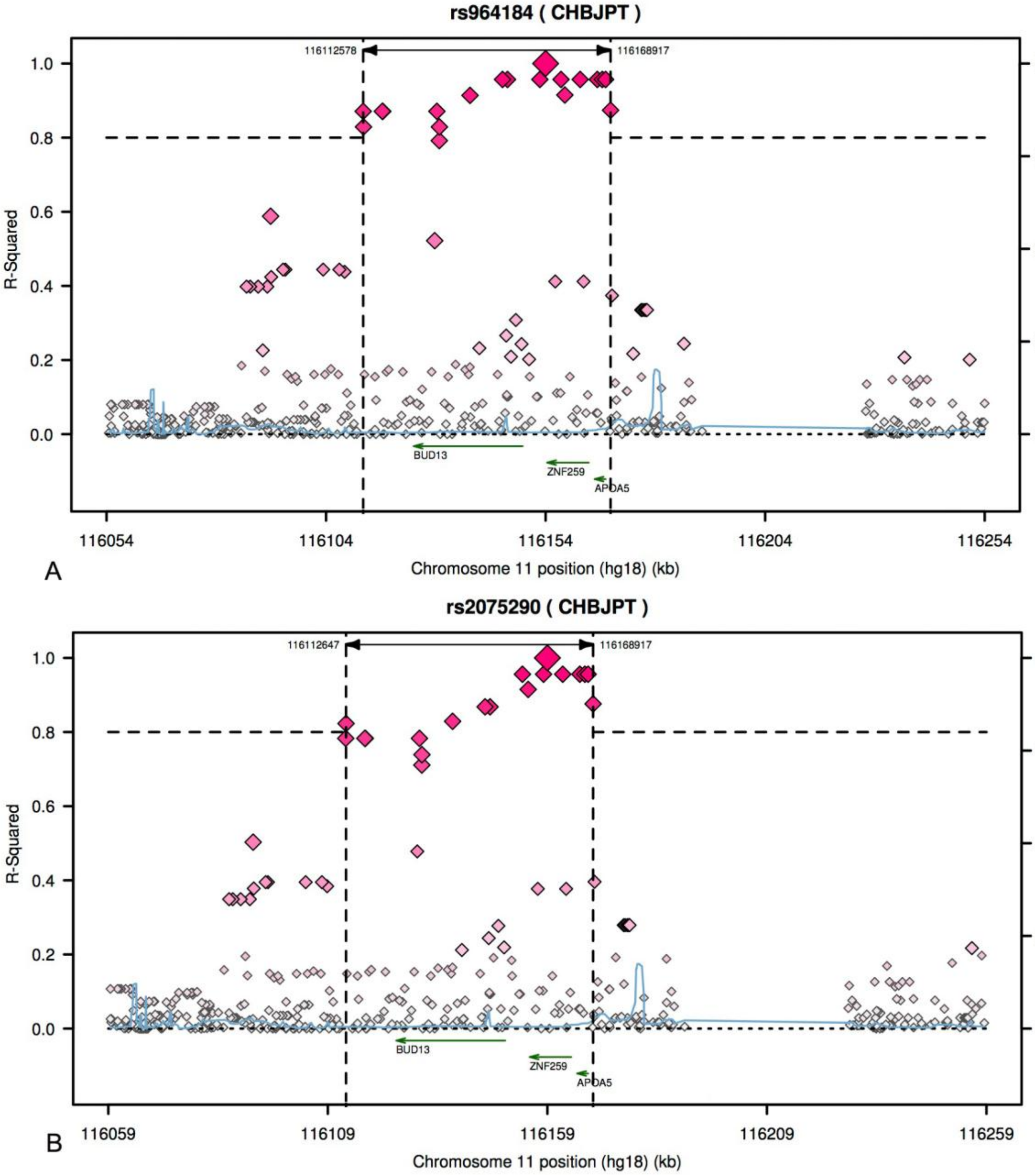

Figure S2 (A) Regional LD plot of rs964184 based on the 1000 Genomes CHB+JPT data. (B) Regional LD plot of rs2075290 based on the 1000 Genomes CHB+JPT data.

The r^2 values of other SNPs versus rs964184 and rs2075290 are indicated by diamond shapes. Recombination rate covering the genomic region of the plot is indicated by the blue line.
